# Production of 2-ketoisocaproate with *Corynebacterium glutamicum* strains devoid of plasmids and heterologous genes

**DOI:** 10.1111/1751-7915.12237

**Published:** 2014-12-09

**Authors:** Michael Vogt, Sabine Haas, Tino Polen, Jan van Ooyen, Michael Bott

**Affiliations:** Institute of Bio- and Geosciences, IBG-1: Biotechnology, Forschungszentrum JülichD-52425, Jülich, Germany

## Abstract

2-Ketoisocaproate (KIC), the last intermediate in l-leucine biosynthesis, has various medical and industrial applications. After deletion of the *ilvE* gene for transaminase B in l-leucine production strains of *Corynebacterium glutamicum*, KIC became the major product, however, the strains were auxotrophic for l-isoleucine. To avoid auxotrophy, reduction of IlvE activity by exchanging the ATG start codon of *ilvE* by GTG was tested instead of an *ilvE* deletion. The resulting strains were indeed able to grow in glucose minimal medium without amino acid supplementation, but at the cost of lowered growth rates and KIC production parameters. The best production performance was obtained with strain MV-KICF1, which carried besides the *ilvE* start codon exchange three copies of a gene for a feedback-resistant 2-isopropylmalate synthase, one copy of a gene for a feedback-resistant acetohydroxyacid synthase and deletions of *ltbR* and *iolR* encoding transcriptional regulators. In the presence of 1 mM l-isoleucine, MV-KICF1 accumulated 47 mM KIC (6.1 g l^−1^) with a yield of 0.20 mol/mol glucose and a volumetric productivity of 1.41 mmol KIC l^−1^ h^−1^. Since MV-KICF1 is plasmid free and lacks heterologous genes, it is an interesting strain for industrial application and as platform for the production of KIC-derived compounds, such as 3-methyl-1-butanol.

## Introduction

*Corynebacterium glutamicum* is the major host for biotechnological production of amino acids, the most important ones being the flavor enhancer l-glutamate and the feed additive l-lysine. In the past decades, *C. glutamicum* strains have been developed for the production of various other commercially interesting compounds (Becker and Wittmann, 2012), including organic acids (Okino *et al*., 2008; Litsanov *et al*., 2012a,b; Wieschalka *et al*., 2013), diamines (Mimitsuka *et al*., 2007; Kind and Wittmann, 2011; Schneider and Wendisch, 2011) or alcohols (Inui *et al*., 2004; Smith *et al*., 2010; Blombach *et al*., 2011). Besides small molecules, also heterologous proteins can be efficiently produced with this Gram-positive bacterium (Scheele *et al*., 2013, and references therein). Thus, *C. glutamicum* has become a production platform in white biotechnology. Three monographs (Eggeling and Bott, 2005; Burkovski, 2008; Yukawa and Inui, 2013) document the rapidly increasing knowledge on this species, which is based on the genome sequence (Ikeda and Nakagawa, 2003; Kalinowski *et al*., 2003) and efficient techniques for its genetic engineering (Kirchner and Tauch, 2003).

**Figure 1:**
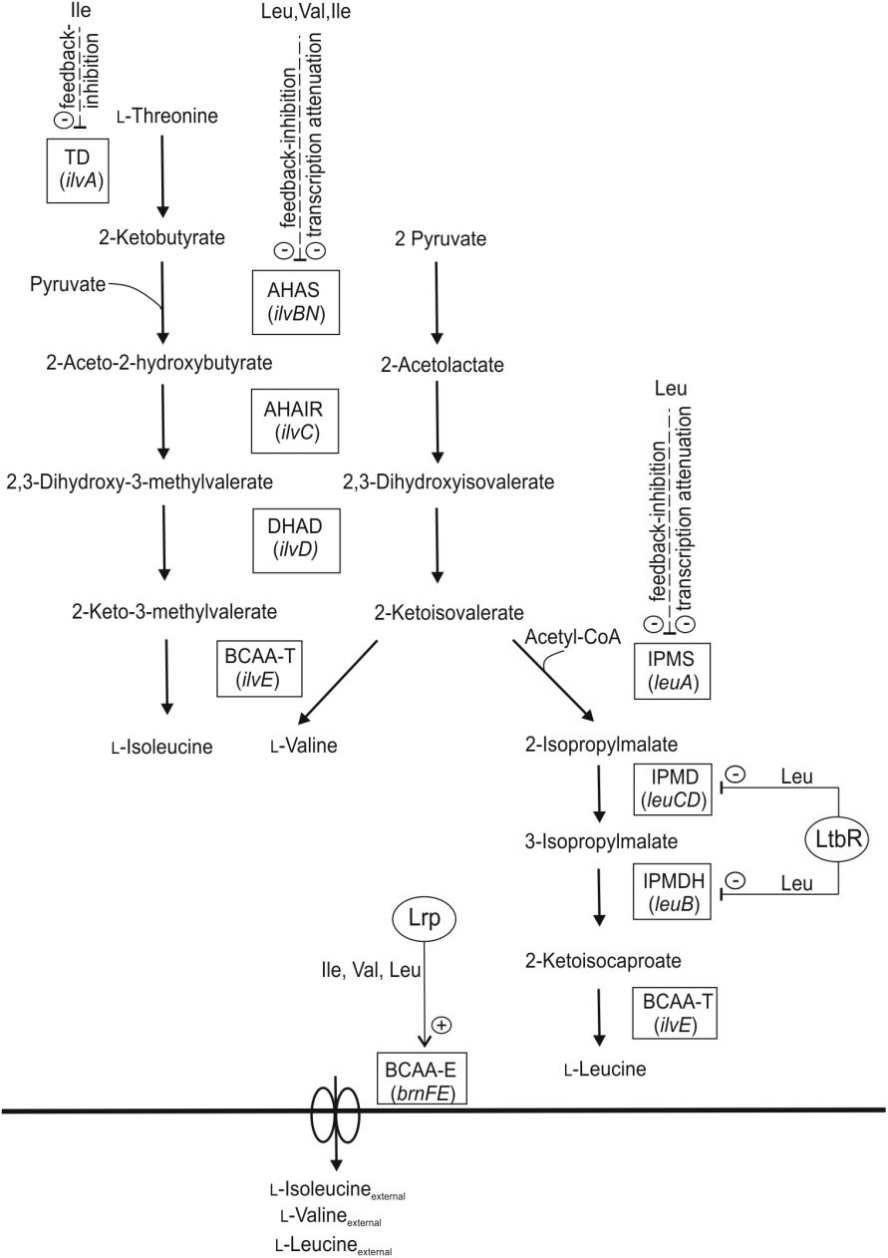
Biosynthesis pathways and their control by various regulatory mechanisms of the three branched-chain amino acids and the respective keto acids in *C. glutamicum*. Enzymes and their corresponding genes are shown in boxes. Lines with ‘+’ indicate activation of gene expression; ‘-’ indicates repression of gene expression (solid lines) or transcription attenuation or feedback inhibition (dashed lines). ‘Leu’, ‘Val’ and ‘Ile’ indicate the presence of l-leucine, l-valine and l-isoleucine respectively. Not shown is the *avtA* gene encoding the branched-chain amino acid transaminase AvtA, which predominantly transaminates 2-ketoisovalerate to l-valine. Abbreviations: AHAIR, acetohydroxyacid isomeroreductase; AHAS, acetohydroxyacid synthase; BCAA-E, branched-chain amino acid exporter (BrnFE); BCAA-T, branched-chain amino acid transaminase IlvE; DHAD, dihydroxyacid dehydratase; IPMD, 3-isopropylmalate dehydratase; IPMDH, 3-isopropylmalate dehydrogenase; IPMS, 2-isopropylmalate synthase; Lrp, leucine-responsive regulatory protein; LtbR, leucine and tryptophane biosynthesis regulator; TD, threonine dehydratase (threonine ammonia-lyase).

The spectrum of amino acids produced with *C. glutamicum* includes the essential branched-chain amino acids (BCAAs) l-valine, l-isoleucine and l-leucine, which are produced in quantities of up to 5000 tons per year in a steadily growing market (Becker and Wittmann, 2012). They have different applications in the food, feed and pharmaceutical industry (Park and Lee, 2010). The biosynthesis pathways of the BCAAs in *C. glutamicum* are overlapping and partly share the same precursors and enzymes (Fig. 1). The direct precursors of l-valine, l-isoleucine and l-leucine are 2-ketoisovalerate (KIV), 2-keto-3-methylvalerate (KMV), and 2-ketoisocaproate (KIC) respectively. These keto acids are predominantly transaminated to the respective amino acids by the transaminase IlvE (Radmacher *et al*., 2002; Marienhagen *et al*., 2005). Similar to their corresponding amino acids, KIV, KMV and KIC have a variety of applications in the medical, biological and food area, since they play an important role in living organisms as regulatory factors in metabolism and key intermediates in biosynthesis (Krause *et al*., 2010; Zhu *et al*., 2011; Bückle-Vallant *et al*., 2014). They are used, for example, in the therapy of chronic kidney disease patients (Aparicio *et al*., 2012). Similar to l-leucine, KIC has anti-catabolic properties through inhibition of muscle proteolysis and provokes enhancement of protein synthesis, especially in the skeletal muscle (Escobar *et al*., 2010; Zanchi *et al*., 2011). Additionally, an insulin-releasing action of KIC (Heissig *et al*., 2005) and an inhibitory effect on glucagon release (Leclercq-Meyer *et al*., 1979) were discussed. It has been shown that KIC can also serve as a basis for the production of the biofuel isopentanol (Cann and Liao, 2010).

KIV, KMV and KIC are mainly produced by chemical synthesis using harsh reaction conditions and multiple purification steps resulting in plenty of waste (Cooper *et al*., 2010). The biotechnological production of these keto acids is thus an interesting alternative. Besides a biotransformation process with *Rhodococcus opacus* using l-leucine as substrate for KIC formation (Zhu *et al*., 2011), fermentative processes with glucose as substrate have recently been described for the production of KIV (Krause *et al*., 2010) and KIC (Bückle-Vallant *et al*., 2014), showing that deletion of *ilvE* in certain engineered *C. glutamicum* strains results in KIC formation. Whereas these strains contained plasmids and in part heterologous genes, the *C. glutamicum* KIC production strains developed in our work are plasmid free and lack heterologous genes.

## Results and discussion

### Initial studies on KIC production using plasmid-containing strains of C. glutamicum

Based on recently developed efficient production strains of *C. glutamicum* ATCC 13032 (Abe *et al*., 1967) for l-leucine (Vogt *et al*., 2014), we intended to modify these strains for the production of KIC. The conversion of KIC to l-leucine is catalysed by the transaminase IlvE, which also converts KIV to l-valine and KMV to l-isoleucine using l-glutamate as amino donor (Radmacher *et al*., 2002; Marienhagen *et al*., 2005). An *ilvE* deletion has been reported to cause auxotrophy for l-leucine and l-isoleucine, but not for l-valine, since the transaminase AvtA also effectively converts KIV to l-valine using l-alanine as amino donor (Marienhagen *et al*., 2005). According to this knowledge, deletion of *ilvE* in l-leucine production strains should lead to the accumulation of KIC and potentially also KMV. In a first series of experiments, we deleted *ilvE* in the wild-type *C. glutamicum* ATCC 13032 and transformed the Δ*ilvE* mutant with plasmid pAN6-*leuA*_B018, carrying an IPTG-inducible *leuA* allele encoding a feedback-resistant 2-isopropylmalate synthase (IPMS) (Vogt *et al*., 2014). 2-Isopropylmalate synthase of *C. glutamicum* is strongly inhibited by l-leucine with a *K*_i_ of 0.4 mM (Pátek *et al*., 1994) and the presence of a feedback-resistant variant is the key for l-leucine overproduction (Vogt *et al*., 2014). The Δ*ilvE* mutant and the Δ*ilvE* strain with plasmid pAN6-*leuA*_B018 were cultivated in 500 ml baffled Erlenmeyer flasks with 50 ml CGXII minimal medium (Keilhauer *et al*., 1993) with 4% (w/v) glucose, 1 mM l-leucine and 1 mM l-isoleucine at 30°C and 120 rpm on a rotary shaker. Keto acids and amino acids were quantified by high-performance liquid chromatography as described (Vogt *et al*., 2014). Chromosomal in-frame deletions and integrations of DNA fragments were performed by two-step homologous recombination using the vector pK19*mobsacB* (Schäfer *et al*., 1994) and a method described previously (Niebisch and Bott, 2001).

*Corynebacterium glutamicum ΔilvE* exhibited a growth rate of 0.38 ± 0.01 h^−1^ and excreted up to 5 mM KIV but no detectable concentrations of KIC (detection limit < 0.1 mM), whereas *C. glutamicum* Δ*ilvE* pAN6-*leuA*_B018 showed a growth rate of 0.30 ± 0.01 h^−1^ and accumulated 37 ± 0.7 mM KIC in the supernatant when induced with 0.1 mM IPTG, confirming that overexpression of the *leuA* allele encoding the feedback-resistant IPMS increased metabolic flux into the leucine pathway (Fig. 1). Surprisingly, *C. glutamicum* Δ*ilvE* carrying pAN6-*leuA*_B018 also accumulated l-leucine (12.3 ± 0.4 mM) and in fact was only auxotrophic for l-isoleucine, but not for l-leucine. A possible limitation of l-valine due to a high metabolic flux from KIV towards KIC was excluded for this strain since additional supplementation of l-valine did not improve growth (data not shown). Accumulation of l-leucine was also reported for other KIC-producing Δ*ilvE* strains and explained by the activity of unspecific transaminases (e.g. AlaT or AvtA) using KIC as substrate when it is present in high concentrations (Bückle-Vallant *et al*., 2014). Consequently, supplementation of the medium with l-leucine was omitted in the following cultivations. The formation of l-leucine as by-product additionally necessitates the presence of feedback-resistant IPMS for KIC overproduction. The results described above demonstrated that our previously described l-leucine producers (Vogt *et al*., 2014) can serve as basis for the construction of KIC production strains.

### Deletion of ilvE in plasmid-free l-leucine production strains

Analogous to our strategy used for l-leucine strain development (Vogt *et al*., 2014), we intended to construct KIC production strains devoid of plasmids, heterologous genes and auxotrophies. Depending on the composition of the medium used in the fermentation process, auxotrophies can necessitate the addition of supplements, increasing the costs of the fermentation process. Plasmids usually necessitate the addition of antibiotics to the medium, which is undesirable for production strains applied in the food and feed industry and can be prohibited by regulatory authorities (Tauch *et al*., 2002). Moreover, the absence of plasmids, antibiotic resistance markers and heterologous genes often results in more stable producer strains (Pátek, 2007). The use of heterologous genes is also an undesired trait for strains used in the food and feed industry. In a first attempt to construct a plasmid-free KIC producer, we deleted the *ilvE* gene in the previously constructed l-leucine producer MV-Leu20 (Table 1; Vogt *et al*., 2014), which contains a deletion of the *ltbR* gene, encoding a repressor of the l-leucine biosynthesis genes, and a replacement of the wild-type *leuA* gene by the feedback-resistant variant *leuA*_B018 under control of the strong *tuf* promoter (Vogt *et al*., 2014). In shake flask cultivations with CGXII medium containing 4% (w/v) glucose, MV-Leu20 accumulated about 20 mM l-leucine. When cultivated in the same medium supplemented with 1 mM l-isoleucine, the strain MV-Leu20 Δ*ilvE* accumulated 18.0 ± 1.6 mM KIC in the supernatant and formed as by-products 5.6 ± 0.3 mM l-leucine, 2.1 ± 0.5 mM KIV and 7.3 ± 1.7 mM KMV. Since KIV and KMV are substrates of the transaminase IlvE (Marienhagen *et al*., 2005), the *ilvE* deletion leads to an accumulation of these keto acids. The presumably low concentrations of l-isoleucine and l-valine in strain MV-Leu20 Δ*ilvE* may also contribute to overproduction of KIV and KMV by reducing the feedback-inhibition of threonine dehydratase (encoded by *ilvA*) by l-isoleucine (Möckel *et al*., 1992) and of acetohydroxyacid synthase (encoded by *ilvBN*) by l-valine and l-isoleucine (Eggeling *et al*., 1987).

**Table 1:** Strains and plasmids used in this study^a^^b^

Strain or plasmid	Relevant characteristics^c^	Source or reference
*C. glutamicum* strains		
Wild type	ATCC 13032, biotin-auxotrophic	Abe and colleagues (1967)
Δ*ilvE*	ATCC 13032 derivative with in-frame deletion of *ilvE*	Marienhagen and colleagues (2005)
MV-Leu20	Rationally designed *C. glutamicum* l-leucine producer (Δ*ltbR* Δ*leuA*::P*tuf*-*leuA*_B018)	Vogt and colleagues (2014)
MV-Leu20 Δ*ilvE*	MV-Leu20 derivative with in-frame deletion of *ilvE*	This study
SH-KIC20	MV-Leu20 derivative with chromosomal replacement of ATG start codon of *ilvE* by GTG start codon	This study
MV-LeuF1	Rationally designed *C. glutamicum* l-leucine producer (Δ*ltbR*::P*tuf*-*leuA*_B018 Δ*leuA*::P*tuf*-*leuA*_B018 IR(cg1121/1122)::P*tuf*-*leuA*_B018 Δ*iolR ilvN*_fbr)	Vogt and colleagues (2014)
MV-KICF1	MV-LeuF1 derivative with chromosomal replacement of ATG start codon of *ilvE* by GTG start codon	This study
Δ*ilvE* Δcg0018	Δ*ilvE* derivative with cg0018 in-frame deletion	This study
Δ*ilvE* Δcg1121	Δ*ilvE* derivative with cg1121 in-frame deletion	This study
Δ*ilvE* Δcg1219	Δ*ilvE* derivative with cg1219 in-frame deletion	This study
Δ*ilvE* Δcg1419	Δ*ilvE* derivative with cg1419 in-frame deletion	This study
Δ*ilvE* Δcg1658	Δ*ilvE* derivative with cg1658 in-frame deletion	This study
Δ*ilvE* Δcg2557	Δ*ilvE* derivative with cg2557 in-frame deletion	This study
Δ*ilvE* Δcg2676	Δ*ilvE* derivative with cg2676 in-frame deletion	This study
Δ*ilvE* Δcg3334	Δ*ilvE* derivative with cg3334 in-frame deletion	This study
Δ*ilvE* Δcg1121::cg1121	Δ*ilvE* Δcg1121 derivative with re-integrated gene cg1121 into its wild-type locus	This study
*E. coli* strains		
DH5α	F^−^ Φ80*lac*ZΔM15 Δ(*lac*ZYA-*arg*F)U169 *rec*A1 *end*A1 *hsd*R17 (r_K_^−^, m_K_^+^) *pho*A *sup*E44 λ– *thi*-1 *gyr*A96 *rel*A1	Invitrogen (Karlsruhe, Germany)
Plasmids		
pAN6	Kan^r^; *E. coli*/*C. glutamicum* shuttle vector for inducible gene expression (P*_tac_*, *lacI*^q^, pBL1 pUC18 *oriV_E.coli_*, pBL1 *oriV_C.glutamicum_*)	Frunzke and colleagues (2008)
pAN6-*leuA*_B018	Kan^r^; pAN6 derivative containing *leuA* allele coding for feedback-resistant 2-isopropylmalate synthase under control of the *tac* promoter	Vogt and colleagues (2014)
pAN6-*leuA*_B018-cg1121	Kanr; pAN6-*leuA*_B018 derivative carrying additional gene coding for cg1121 along with its upstream (94 bp) and downstream (305 bp) regions	This study
pK19*mobsacB*	Kan^r^; vector for allelic exchange in *C. glutamicum* (pK18 *oriV_E.coli_ sacB lacZ*α)	Schäfer and colleagues (1994)
pK19*mobsacB*-Δ*ilvE*	Kan^r^, pK19*mobsacB* derivative for in-frame deletion of gene *ilvE*	Marienhagen and colleagues (2005)
pK19*mobsacB*-GTG-*ilvE*	Kan^r^, pK19*mobsacB* derivative for replacement of ATG start codon of *ilvE* by GTG	This study
pK19*mobsacB*-Δcg0018	Kan^r^, pK19*mobsacB* derivative for in-frame deletion of gene coding for Cg0018	This study
pK19*mobsacB*-Δcg1121	Kan^r^, pK19*mobsacB* derivative for in-frame deletion of gene coding for Cg1121	This study
pK19*mobsacB*-Δcg1219	Kan^r^, pK19*mobsacB* derivative for in-frame deletion of gene coding for Cg1219	This study
pK19*mobsacB*-Δcg1419	Kan^r^, pK19*mobsacB* derivative for in-frame deletion of gene coding for Cg1419	This study
pK19*mobsacB*-Δcg1658	Kan^r^, pK19*mobsacB* derivative for in-frame deletion of gene coding for Cg1658	This study
pK19*mobsacB*-Δcg2557	Kan^r^, pK19*mobsacB* derivative for in-frame deletion of gene coding for Cg2557	This study
pK19*mobsacB*-Δcg2676	Kan^r^, pK19*mobsacB* derivative for in-frame deletion of gene coding for Cg2676	This study
pK19*mobsacB*-Δcg3334	Kan^r^, pK19*mobsacB* derivative for in-frame deletion of gene coding for Cg3334	This study
pK19*mobsacB*-cg1121	Kan^r^, pK19*mobsacB* derivative for re-integration of gene coding for Cg1121 into its wild-type locus	This study

a All constructed plasmids as well as chromosomal deletions and integrations in engineered strains were verified by DNA sequencing.

b Plasmid constructions were performed in *E. coli* DH5α. Description of plasmid constructions and used DNA oligonucleotides (Table S1) can be found in the Supporting Information.

c Kan^r^, kanamycin resistance

As mentioned above, l-leucine formation in the absence of the transaminase IlvE is presumably due to high cytoplasmic KIC concentrations, allowing its conversion by other transaminases such as AvtA that have weak affinities for KIC (Marienhagen *et al*., 2005). To test this assumption, we measured the cytoplasmic KIC concentrations in the wild type and strain MV-Leu20 Δ*ilvE*. Cells were grown in CGXII medium with 4% (w/v) glucose, harvested in the early exponential phase (optical density at 600 nm (OD_600_ = 5), and cytoplasmic concentrations were determined as described (Paczia *et al*., 2012). The internal KIC concentration of the wild type was below the detection limit of 5 μM, whereas MV-Leu20 Δ*ilvE* accumulated approximately 2.8 mM KIC inside the cell, corresponding to a more than 500-fold increase.

### Exchange of the ilvE start codon in plasmid-free l-leucine production strains

**Figure 2:**
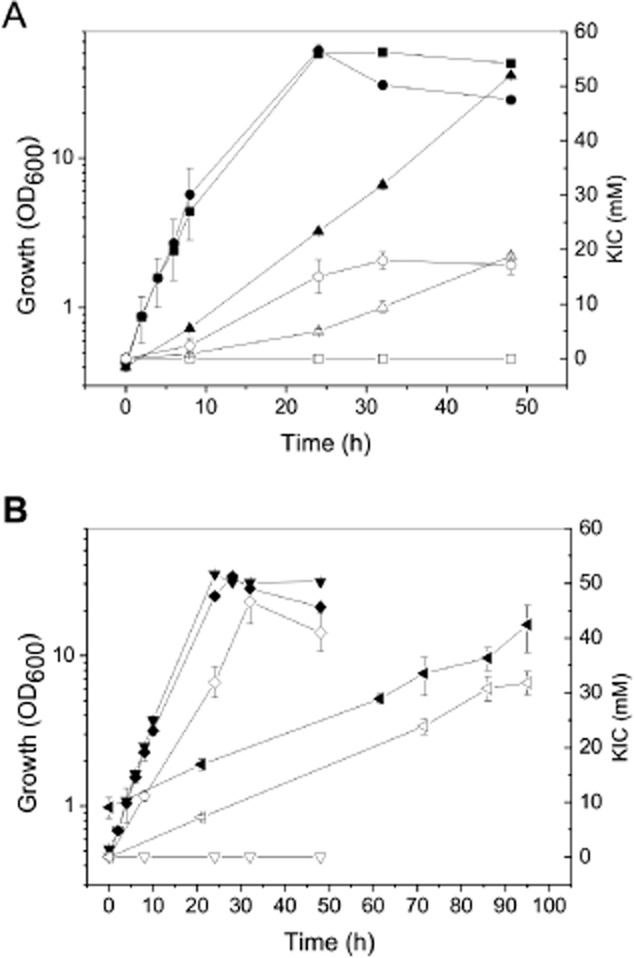
Growth and KIC formation of different *C. glutamicum* strains in shake flasks with CGXII minimal medium containing 4% (w/v) glucose.A. MV-Leu20 without supplements (growth, ▪; KIC, □), MV-Leu20 Δ*ilvE* supplemented with 1 mM l-isoleucine (growth, •; KIC, ○) and SH-KIC20 without supplements (growth, ▴; KIC, △).B. MV-LeuF1 without supplements (growth, ▾; KIC, ▽), MV-KICF1 without supplements (growth, ◂; KIC, ◃) and MV-KICF1 with 1 mM l-isoleucine (growth, ♦; KIC, ⋄). The data represent mean values and standard deviations obtained from three independent cultivations.

To avoid the l-isoleucine auxotrophy of strain MV-Leu20 Δ*ilvE*, we intended to reduce IlvE activity to a value that was high enough to provide l-isoleucine for growth, but low enough to allow KIC overproduction. For this purpose, the ATG start codon of *ilvE* was exchanged against GTG, which should decrease the translation rate (Becker *et al*., 2010) of the *ilvE* transcript and thereby reduce the specific IlvE activity. The start codon exchange was performed in the l-leucine producers MV-Leu20 and MV-LeuF1 (Table 1). The latter strain contains (i) three copies of the *leuA*_B018 gene in the chromosome under control of the *tuf* promoter, two of them replacing *ltbR* and the native *leuA* gene, (ii) a deletion of *iolR* (Klaffl *et al*., 2013) for enhanced glucose uptake and (iii) a feedback-resistant acetohydroxyacid synthase encoded by *ilvN*_fbr (Vogt *et al*., 2014). The strains SH-KIC20 (from MV-Leu20) and MV-KICF1 (from MV-LeuF1) resulting from the *ilvE* start codon exchange (Table 1) were cultivated in CGXII medium with 4% (w/v) glucose to test for KIC accumulation in the supernatant (Fig. 2).

Without supplementation of l-isoleucine, SH-KIC20 showed a strongly reduced growth rate of 0.08 ± 0.01 h^−1^ compared with the ancestor strain MV-Leu20 (0.31 ± 0.01 h^−1^). This phenotype suggests that the *ilvE* start codon exchange reduced the availability of BCAAs and consequently the growth rate. Strain SH-KIC20 accumulated about 19 mM KIC in 49 h, which correlates with the KIC concentration produced by MV-Leu20 Δ*ilvE*. The slower growth of SH-KIC20 led to a lowered volumetric productivity of 0.38 mmol l^−1^ h^−1^ in comparison to MV-Leu20 Δ*ilvE* (approximately 0.8 mmol l^−1^ h^−1^). Strain MV-KICF1 showed a growth rate of only 0.03 ± 0.01 h^−1^, which is 85% lower than the one of the parent strain (μ = 0.20 ± 0.01 h^−1^), and yielded a maximal KIC concentration of 33 mM after 95 h (Table 2). Supplementation of the medium with 1 mM l-isoleucine enabled MV-KICF1 to reach the same growth rate (μ = 0.21 ± 0.01 h^−1^) as its parent MV-LeuF1 and to form 47 ±q 4 mM KIC (6.1 g l^−1^) after 32 h with a yield of 0.20 ± 0.02 mol KIC per mol of glucose and a productivity of 1.41 ± 0.13 mmol KIC l^−1^ h^−1^ (Table 2). Both SH-KIC20 and MV-KICF1 formed KIV, KMV and l-leucine as by-products (Table 2).

**Table 2:** Growth and production parameters of strains MV-KICF1 and SH-KIC20 in shake flask cultivations^a^^b^

Parameter	MV-KICF1 + 1 mM l-isoleucine	MV-KICF1 without l-isoleucine	SH-KIC20^d^ without l-isoleucine
Growth rate (h^−1^)	0.21 ± 0.01	0.03 ± 0.01	0.08 ± 0.01
KIC (mM)	46.7 ± 4.1	31.8 ± 2.1	18.8 ± 0.67
By-products^c^:			
KIV (mM)	13.3 ± 2.2	19.0 ± 4.1	2.6 ± 0.3
KMV (mM)	8.8 ± 1.3	4.9 ± 0.6	8.7 ± 0.1
l-leucine (mM)	3.0 ± 0.2	10.3 ± 3.1	4.8 ± 0.2
Molar product yield (mol KIC per mol glucose)	0.204 ± 0.018	0.143 ± 0.010	0.084 ± 0.001
Volumetric productivity (mmol KIC l^−1^ h^−1^)	1.41 ± 0.13	0.34 ± 0.02	0.38 ± 0.02

a Cultivations were performed in 500 ml baffled shake flasks containing 50 ml CGXII minimal medium with 4% (w/v) glucose. Supplementation of l-isoleucine is indicated.

b Mean values and standard deviations from three independent cultivations are shown.

c Concentrations of l-valine and l-isoleucine were below 2 mM.

d Cultivation of SH-KIC20 supplemented with 1 mM l-isoleucine was not tested.

The results described above demonstrate that the start codon exchange for reduction of IlvE activity was successful and allowed growth and KIC accumulation without supplementation of BCAAs; however, the production parameters were lower compared with supplementation with 1 mM l-isoleucine (Table 2). A successful industrial application depends on high product yields combined with sufficient cell growth, resulting in competitive productivity. Therefore, the addition of l-isoleucine is still important to improve growth of the constructed strains to reach better productivity values. As an alternative approach to adjust the IlvE activity to an optimal value for prototrophic growth with simultaneous KIC production, *ilvE* gene expression could be fine-tuned by testing promoters with varying strength (Vašicová *et al*., 1999; Hammer *et al*., 2006).

Recently, Bückle-Vallant and colleagues (2014) described a plasmid-based *C. glutamicum* strain for the production of KIC. This strain is characterized by deletions of the genes *ilvE*, *ltbR*, *prpC1* and *prpC2*, an exchange of the two *gltA* promoters (van Ooyen *et al*., 2011) by the mutated *dapA* promoter L1 (Vašicová *et al*., 1999) to reduce citrate synthase activity (van Ooyen *et al*., 2012), and plasmid-based overexpression of *ilvBNCD* and a *leuA* allele of *Escherichia coli* encoding a feedback-resistant IPMS. This strain accumulated up to 71 mM KIC when cultivated with glucose plus acetate as carbon sources and supplemented with 2 mM each of l-isoleucine and l-valine. Under cultivation conditions comparable to ours, i.e. without acetate, this strain reached KIC titres (54 ± 4 mM) and yields (0.22 mol per mol of glucose) in a similar range as strain MV-KICF1 when supplemented with l-isoleucine. In comparison, the biotransformation with *Rhodococcus opacus* transcribed by Zhu and colleagues (2011) reached about 10 mM KIC using 39 mM l-leucine as substrate.

### 2-Ketoisocaproate transport

When MV-KICF1 was batch cultivated in a bioreactor as described (Vogt *et al*., 2014) using a medium supplemented with 1 mM l-isoleucine, comparable KIC titres as in shake flasks of about 50 mM were achieved (data not shown). Interestingly, additional feeding of glucose in fed-batch experiments did not further increase the KIC concentrations and led to an arrest of cell growth and glucose consumption (data not shown). A possible explanation is a block of the l-leucine biosynthesis pathway by elevated KIC concentrations, as Bückle-Vallant and colleagues (2014) found competitive inhibition of IPMS by KIC and non-competitive inhibition of 3-isopropylmalate dehydratase by KIC. Additionally, a competitive inhibition of acetohydroxyacid synthase by KIV has been reported by Krause and colleagues (2010). To avoid a reduced flux into the leucine synthesis pathway by competitive inhibition of IPMS by KIC, the concentration of KIV within the cell needs to be increased and/or the concentration of KIC within the cell should be decreased. These concentrations are determined on one hand by the rates of synthesis and further metabolic conversion and on the other hand by the rates of export to and import from the supernatant.

Knowledge on KIC transport is very limited. Obviously, as shown by our studies and that of Bückle-Vallant and colleagues (2014), KIC can leave the cell, and previous studies by Groeger and Sahm (1987) demonstrated that KIC can enter the cell. As previously described for l-isoleucine (Zittrich and Krämer, 1994), passive diffusion, carrier-mediated uptake and carrier-mediated excretion must be considered as possibilities for an amphiphilic solute like KIC to cross the cytoplasmic membrane. Due to its similarity to l-isoleucine, it seems likely that also KIC is able to diffuse across the membrane. However, a necessity to possess carriers for KIC import or KIC export is not obvious, in contrast to the advantage of having importers for amino acids and exporters for non-catabolizable amino acids. In *C. glutamicum*, the export of BCAAs and methionine is catalysed by the exporter BrnFE (Kennerknecht *et al*., 2002; Trötschel *et al*., 2005; Xie *et al*., 2012), but evidence is available that BrnFE is not involved in KIC export (Radespiel, 2010). The identification of transporters can be beneficial for biotechnological processes to increase productivity since transport of desired products into the medium often represents a bottleneck. For example, export was identified as a limiting factor for l-isoleucine production with *C. glutamicum* (Morbach *et al*., 1996), and the production of this BCAA was improved by overexpression of the respective transporter encoded by *brnFE* (Kennerknecht *et al*., 2002; Xie *et al*., 2012).

**Figure 3:**
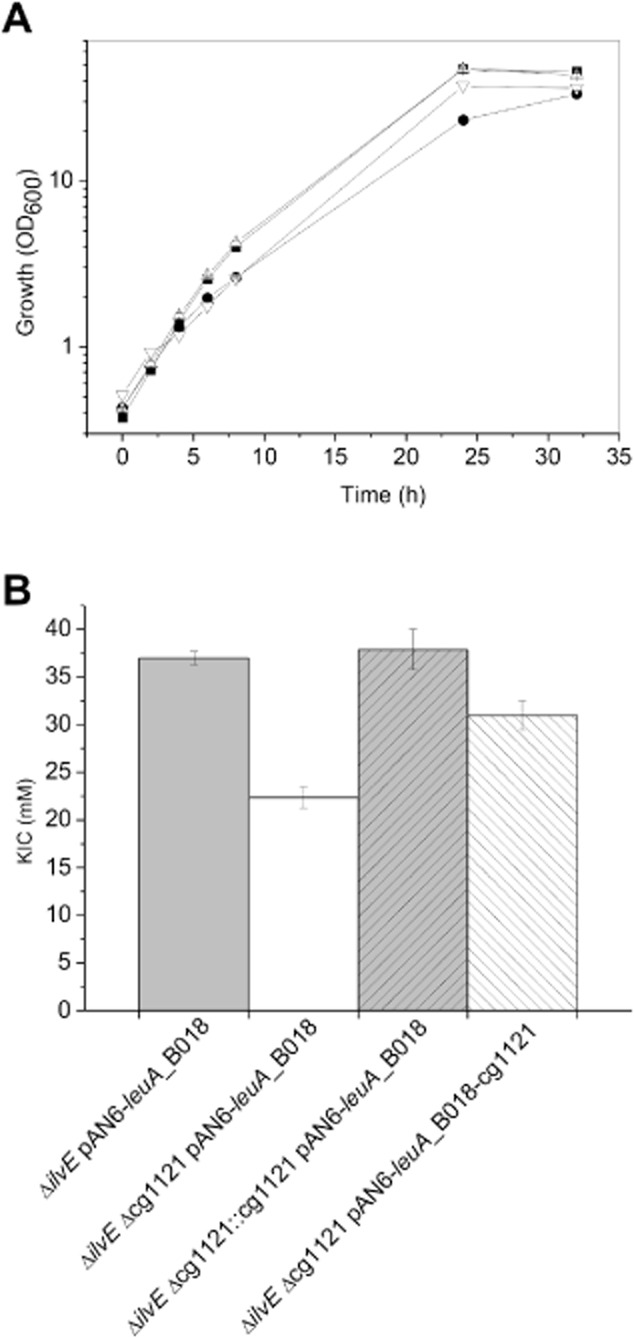
Complementation of the effects on growth and KIC accumulation caused by deletion of cg1121 in strain *C. glutamicum* Δ*ilvE* carrying pAN6-*leuA*_B018.A. Growth of strains Δ*ilvE* with pAN6-*leuA*_B018 (▪), Δ*ilvE* Δcg1121 with pAN6-*leuA*_B018 (•), Δ*ilvE* Δcg1121::cg1121 with pAN6-*leuA*_B018 (△) and Δ*ilvE* Δcg1121 with pAN6-*leuA*_B018-cg1121 (▽) are shown.B. Maximal KIC concentrations reached after 32 h cultivation in 500 ml baffled shake flasks with 50 ml CGXII minimal medium containing 4% (w/v) glucose and 0.1 mM IPTG at 30°C and 120 rpm on a rotary shaker. The deletion of cg1121 was complemented either by genomic reintegration of cg1121 (Δ*ilvE* Δcg1121::cg1121 pAN6-*leuA*_B018) or by plasmid-borne expression of cg1121 (Δ*ilvE* Δcg1121 pAN6-*leuA*_B018-cg1121). The data represent mean values and standard deviations obtained from three independent cultivations.

In order to test if a KIC exporter is present in *C. glutamicum* that might be useful to improve KIC overproduction, we searched for genes showing increased mRNA levels during KIC production by performing comparative transcriptome analyses using DNA microarrays as described previously (Vogt *et al*., 2014). KIC producer strain MV-Leu20 Δ*ilvE* was compared with the wild type to determine differentially expressed genes, resulting in a list of eight candidate transporter genes (Table 3). Each of these genes was deleted in the *C. glutamicum* Δ*ilvE* background, and the resulting double deletion mutants were transformed with pAN6-*leuA*_B018. When cultivated in glucose minimal medium with 0.1 mM IPTG, one of the eight strains, which contained a deletion of cg1121 (annotated as putative permease of the major facilitator superfamily), showed a reduced growth rate (0.23 ± 0.01 h^−1^) and a reduced maximal KIC titre (22.4 mM) compared with the reference strain Δ*ilvE* pAN6-*leuA*_B018 (0.30 ± 0.01 h^−1^, 37 mM KIC). The phenotype could be complemented by reintegration of cg1121 into the genome of strain Δ*ilvE* Δcg1121 or by plasmid-borne expression of cg1121 (Fig. 3). However, the specific KIC export rates (determined as described by Kennerknecht *et al*., 2002) of strain Δ*ilvE* Δcg1121 (6.5 ± 1.0 nmol min^−1^ g_CDW_^−1^) were not significantly reduced compared with that of the reference strain Δ*ilvE* (8.0 ± 1.5 nmol min^−1^ g_CDW_^−1^) and the cytoplasmic KIC concentrations of the two strains at an OD_600_ of 5 were comparable. Therefore, the role of Cg1121 for growth and KIC production remains unclear and needs further investigations.

**Table 3:** Putative transporter genes showing increased expression in a KIC producer

Gene	Annotation	mRNA ratio^a^ (MV-Leu20 *ΔilvE*/ Wild type	TMH^b^
cg0018	putative membrane protein, conserved	3.5	9
cg1121	putative permease of the major facilitator superfamily	2.2	7
cg1219	putative membrane protein	3.5	10
cg1419	putative Na^+^-dependent transporter, bile acid:Na^+^ symporter BASS family	7.2	8
cg1658	putative permease of the major facilitator superfamily	35.6	12
cg2557	putative secondary Na^+^/bile acid symporter, bile acid:Na^+^ symporter BASS family	2.5	8
cg2676	putative ABC-type dipeptide/oligopeptide/nickel transport system, permease component	2.1	6
cg3334	putative arabinose efflux permease, MFS type	2.0	12

a Transcriptome analyses of KIC producer MV-Leu20 Δ*ilvE* in comparison to the wild type were performed using DNA microarrays as described (Vogt *et al*., 2014). Candidate transporter genes were chosen based on an mRNA ratio (MV-Leu20 Δ*ilvE*/wild type) of > 2, an annotation as (putative) membrane or transporter proteins and the prediction of multiple transmembrane helices in the encoded proteins. Data represent mean values of at least two (maximum four) evaluable microarray experiments (*P*-value < 0.05).

b TMH, number of transmembrane helices predicted with the sosui engine version 1.11.

## Supporting Information

**Table S1.** DNA oligonucleotides used in this study.
