# Supplementary material for: Production of 2-ketoisocaproate with Corynebacterium glutamicum strains devoid of plasmids and heterologous genes
Source: Microb Biotechnol. 2014 Dec 9;8(2):351–60. doi: 10.1111/1751-7915.12237 (PMC4353348; doi:10.1111/1751-7915.12237)
Supplement: Supplementary file 1 [file mbt20008-0351-sd1.pdf]

Supporting Information for Vogt *et al.* 2014

Table S1. DNA oligonucleotides used in this study.

Name	DNA Sequence (5´- 3´)
**Oligonucleotides used for construction of plasmid pK19*mobsacB*-GTG-*ilvE***	
pilvE_SH0f_SbfI	AAAACCTGCAGGCCGCAATGACAGGCGCAACGTCG
pilvE_SH0r_XbaI	AAAATCTAGAGTGACCGTCACGTGCGATGAGGCCGTCCATGAG
pilvE_GTGf	GCAGGTGTACCTTAAAATCCGTGACGTCATTAGAG
pilvE_GTGr	CTCTAATGACGTCACGGATTTTAAGGTACACCTGC
**Oligonucleotides used for construction of plasmid pK19*mobsacB*-Δcg0018**	
cg0018_sf	CTATCCGAGGTGGGTGAGTAGGGTGCCCGCGACGATAAGCGGACT CACACCAG
cg0018_sr	AAAAAAGCTTGCCCCAACATGCAATTTCAGCGGCTGC
cg0018_ef	AAAACCTGCAGGCCTCTGAAACACAAACCCCTAGACACTGC
cg0018_er	GGCACCCTACTCACCCACCTCGGATAG
**Oligonucleotides used for construction of plasmid pK19*mobsacB*-Δcg1121**	
cg1121_sf	AAAACCTGCAGGCGCGACCAGCAGCTTGACCAGAGC
cg1121_sr	GCGGTTTTGGAAGTAGCTAAGCAGACGGTCGTCAATCAAGTTAAC GCATCAAGGATC
cg1121_ef	CGTCTGCTTAGCTACTTCCAAAACCGC
cg1121_er	AAAAAAGCTTGAGAGCGTAAGGCCCCTACTTCCTG
**Oligonucleotides used for construction of plasmid pK19*mobsacB*-Δcg1219**	
cg1219_sf	AAAACCTGCAGGCGTTTTCTCCTGGGATGGCTTTCAACGC
cg1219_sr	GGTTTAGATTCTGATCATTGTTACGCCGAGCCCGAATAGCACTGC AAGCATGAGGTTTTC
cg1219_ef	CTCGGCGTAACAATGATCAGAATCTAAACC
cg1219_er	AAAAAAGCTTCCAGTAGAATGCCACATCGTGGAACACGG
**Oligonucleotides used for construction of plasmid pK19*mobsacB*-Δcg1419**	
cg1419_sf	AAAACCTGCAGGGGCTGCTGTATCTGCAACGGCAACC
cg1419_sr	GCCTTCTCCGAGGCAGCCTTTTCAACTTGAGTACTCATGTGCTCCC TAACTG
cg1419_ef	GAAAAGGCTGCCTCGGAGAAGGC
cg1419_sr	AAAAAAGCTTCGCCCTTGGCGTTAACCACAGGAAG

Oligonucleotides used for construction of plasmid pK19*mobsacB*- Δcg1658

cg1658_sf	CTTTTAGACCTCTCGGAGGTCGTGGTTCGTGGAGTGATCAGTTGTGGTCACGAG
cg1658_sr	AAAAAAGCTTGCTGTTGATCTCTCGGGAATGCGTG
cg1658_ef	AAAACCTGCAGGGGTGGGCAAGCACGGTTCCAAGCGG
cg1658_er	CTTTTAGACCTCTCGGAGGTCGTGGTT

Oligonucleotides used for construction of plasmid pK19*mobsacB*-Δcg2557

cg2557_sf	GTTAGCTGGAAACCGCGACGGATTCCTTCGCTGAACGGCCTTCTTCTGTTTTGGACAC
cg2557_sr	AAAAAAGCTTCGGTGTCGTTGTAAGCGCGCAGGG
cg2557_ef	AAAACCTGCAGGGCGCTCCCAAGCGTGCAGGATTTG
cg2557_er	AAGGAATCCGTCGCGGTTTCCAGCTAAC

Oligonucleotides used for construction of plasmid pK19*mobsacB*-Δcg2676

cg2676_sf	GTAGTTGGCAGACATAACCACGGCGATAGGATTACTCATGAGTCTTATGCCCTTTCCC
cg2676_sr	AAAAAAGCTTGGGCATCATCTTGATCCAGATCGTGTCC
cg2676_ef	AAAACCTGCAGGCGCGCTCTAGAAGCTCAATGACCTTC
cg2676_er	ATCGCCGTGGTTATGTCTGCCAACTAC

Oligonucleotides used for construction of plasmid pK19*mobsacB*-Δcg3334

cg3334_sf	AAAACCTGCAGGTGTAATCTGCCGATTCTGTATTTGACGTGC
cg3334_sr	GGGCTGCTACTTCAGTAGCGGGCGGGTGGTGCTCATGGCTGCGAAGCC
cg3334_ef	CGCCCGCTACTGAAGTAGCAGCCC
cg3334_er	AAAAAAGCTTGGTGTTTCCAATGCCCGTGTGGGTGCG

Oligonucleotides used for construction of plasmid pAN6-*leuA*_B018-cg1121

AvrII_cg1121f	GGGGCCTAGGCTGAACACTCCTGTACCTGTACAAATG
AvrII_cg1121r	GGGGCCTAGGGGCGATTCTGAGAAGCCATTTTC

Plasmid constructions

Overlap-extension PCR was applied for the construction of vector pK19*mobsacB*-GTG-*ilvE*: The upstream region of *ilvE* (approximately 500 bp) and *ilvE* along with its downstream region (approximately 1600 bp) were amplified via the oligonucleotide pairs pilvE_SH0f_SbfI/ pilvE_GTGr and pilvE_GTGf/pilvE_SH0r_XbaI, respectively, from genomic DNA of *C. glutamicum* wild type. The amplified DNA fragments were fused in a PCR reaction via overlapping regions that introduce a mutation into *ilvE* leading to the start codon exchange ATG->GTG. This fusion product (approximately 2100 bp) was amplified using oligonucleotides pilvE_SH0f_SbfI and pilvE_SH0r_XbaI, digested with the restriction endonucleases *Sbf*I and *Xba*I and finally ligated into the likewise digested vector pK19*mobsacB*.

To obtain plasmid pK19*mobsacB*-Δcg1121, the regions upstream and downstream (each 500 bp, approximately) of the gene coding for Cg1121 were amplified via the oligonucleotide pairs cg1121_sf/cg1121_sr and cg1121_ef/cg1121_er, respectively, from genomic DNA of *C. glutamicum* wild type. The amplified DNA fragments were fused in an overlap-extension PCR resulting in a product (approximately 1000 bp) that was amplified with oligonucleotides cg1121_sf and cg1121_er. This PCR fragment was digested with the restriction enzymes *Sbf*I and *Hind*III and ligated into the likewise digested vector pK19*mobsacB*.

The vectors for deletion of the genes cg0018, cg 1219, cg 1419, cg1658, cg2557, cg2676, and cg3334 (Table 1) were constructed analogously to plasmid pK19*mobsacB*-Δcg1121 via overlap-extension PCR, using the respective primers (Table S1). Amplified fused PCR products were digested with the restriction enzymes *Sbf*I and *Hind*III and ligated into the likewise digested vector pK19*mobsacB.*

To obtain vector pK19*mobsacB*-cg1121, the gene coding for Cg1121 (861 bp) along with its upstream and downstream regions (each 500 bp, approximately) was amplified via the oligonucleotide pairs cg1121_sf and cg1121_er from genomic DNA of *C. glutamicum* wild type. The resulting PCR product (approximately 1900 bp) was digested with the restriction enzymes *Sbf*I and *Hind*III and ligated into the likewise digested vector pK19*mobsacB*.

The plasmid pAN6-*leuA*_B018-cg1121 was constructed by amplifying the coding sequence for cg1121 (861 bp) along with its upstream (94 bp) and downstream (305 bp) regions from genomic DNA of *C. glutamicum* wild type via the oligonucleotides AvrII_cg1121f and AvrII_cg1121r. The PCR fragment was digested with the restriction enzyme *Avr*II and ligated into the likewise digested vector pAN6-*leuA*_B018.
